# PR3-ANCA in Wegener's granulomatosis prime human mononuclear cells for enhanced activation via TLRs and NOD1/2

**DOI:** 10.1186/1746-1596-4-23

**Published:** 2009-07-14

**Authors:** Akiko Uehara, Tadasu Sato, Atsushi Iwashiro, Sou Yokota

**Affiliations:** 1 Department of Microbiology and Immunology, Tohoku University Graduate School of Dentistry, Sendai, Japan

## Abstract

**Background:**

Anti-neutrophil cytoplasmic antibodies (ANCA) is autoantibodies characteristic of vasculitis diseases. A connection between ANCA and Wegener's granulomatosis was well established. The interaction of both ANCA phenotypes (PR3-ANCA and MPO-ANCA) with leukocytes provoked cell activation, which might be involved in the pathogenesis of ANCA-related Wegener's granulomatosis.

**Methods:**

In this study, we examined whether PR3-ANCA sera and purified immunoglobulins from patients with Wegener's granulomatosis prime human monocytic cells for enhanced responses to microbial components in terms of production of proinflammatory cytokines.

**Results:**

Flow cytometry demonstrated that stimulation with antibodies to proteinase 3 enhanced the expression of TLR2, 3, 4, 7, and 9, NOD1, and NOD2 in human mononuclear cells. The sera and purified immunoglobulins significantly primed human mononuclear cells to secrete interleukin-8 in response to microbial components via TLRs and NODs. Priming effects were also observed for the production of interleukin-6, monocyte chemoattractant protein-1, and tumor necrosis factor-α. On the other hand, PR3-ANCA-negative sera from patients with polyarteritis nodosa which possibly related to MPO-ANCA and aortitis syndrome as well as control sera from a healthy volunteer did not have any priming effects on PBMCs.

**Conclusion:**

In conclusion, PR3-ANCA prime human mononuclear cells to produce cytokines upon stimulation with various microbial components by up-regulating the TLR and NOD signaling pathway, and these mechanisms *may partially *participate in the inflammatory process in Wegener's granulomatosis.

## Background

Anti-neutrophil cytoplasmic antibodies (ANCA) form a heterogeneous group of Abs that target antigens present mostly in azurophilic granules of polymorphonuclear leukocytes. ANCA were first discovered in the 1970s, when cytoplasmic fluorescence was observed during investigations of anti-nuclear antibodies (Abs) by indirect fluorescence on human granulocytes [1]. In the 1980s, the spectrum of diseases associated with ANCA became clearer, and vasculitis [2,3] was identified as a common sign of these diseases. A connection between ANCA and Wegener's granulomatosis (WG) was established [4].

ANCA are directed against antigens located in the cytoplasmic space of polymorphonuclear leukocytes and monocytes. Two of the main targets have been identified as enzymes, both part of the azurophilic granules of neutrophils; these enzymes are proteinase 3 (PR3) [5] and myeloperoxidase (MPO) [6]. The antigenic specificity of ANCA may be illustrated by immunofluorescent labeling, during an investigation of ANCA on ethanol-fixed granulocytes. The cytoplasmic fluorescence forms a characteristic picture of PR3-ANCA and is mainly associated with reactivity to PR3, whereas perinuclear fluorescence reflects MPO-ANCA, the target for which is represented, in most cases, by MPO [7].

ANCA have been reported to be causally involved in the pathogenesis of WG; the autoantibody titer correlates with disease activity [8], and ANCA directly activate a wide variety of inflammatory functions in neutrophils, such as the secretion of oxygen radicals, proteases, and lipid mediators, once PR3 is expressed on the leukocyte surface [9-12] under inflammatory conditions. Additionally, in isolated monocytes, anti-PR3 Abs stimulate the release of proinflammatory cytokines [13,14]. Nowack et al. [15] reported that the expression of CD14 and CD18 was up-regulated on monocytes by ANCA as well as monoclonal Abs against PR3 in vitro. Anti-PR3 Abs provoked a marked release of cytokines in human monocytes with the early appearance of tumor necrosis factor (TNF)-α and interlekin (IL)-1β and the delayed release of IL-6, IL-8, and thromboxane A_2 _[16]. In addition, anti-PR3 Abs induced the release of monocyte chemoattractant protein (MCP)-1 from human mononuclear cells [17]. Hatter et al. [18] reported that PR3 was detected in human renal tubular epithelial cells treated with TNF-α, and the primed cells responded to anti-PR3 Abs with the activation of a phosphoinositide-related signal transduction pathway. Recently, Bartùòková et al. [19] reported that the interaction of PR3-ANCA with TNF-α-primed mononuclear cells stimulated the release of IL-8 via cross-linking between Fc gamma receptors and PR3 expressed on the monocyte cell surface. More recently, Hattar et al. [20] demonstrated a priming effect of PR3-ANCA for the activation of isolated monocytes and neutrophils by bacterial cell-surface components such as lipopolysaccharide (LPS) and lipoteichoic acid. Although the incubation of monocytes and neutrophils with ANCA alone resulted in only a low level of IL-8 release, preincubation with ANCA, but not with isotype-matched control immunoglobulin (Ig)G, resulted in a markedly enhanced release of IL-8 upon stimulation with LPS. ANCA-related priming was also observed for the production of TNF-α and IL-6. Flow cytometric analysis revealed an increase in the expression of CD14 on monocytes and neutrophils following the priming with ANCA. Therefore, they concluded that ANCA specifically prime monocytes and neutrophils in a CD14-dependent manner, and the resulting enhanced responsiveness to bacterial components may contribute to the development and maintenance of inflammatory lesions during WG.

The innate immune system recognizes microorganisms through a series of pattern recognition receptors that are highly conserved in evolution, specific for common motifs found in microorganisms but not in eukaryotes, and designated as pathogen-associated molecular patterns (PAMPs) [21-23]. Representative microbial PAMPs are the lipid A moiety of LPS from Gram-negative bacteria, lipopeptides from various bacteria, including mycoplasma, peptidoglycans (PGNs) from either Gram-positive or Gram-negative bacteria except mycoplasma which are devoid of cell walls and viral double-stranded and single-stranded RNA. Several studies have demonstrated that in mammals, these PAMPs are recognized specifically by the respective Toll-like receptor (TLR) [24]. In addition, NOD-like receptor (NLR) family members were demonstrated to be intracellular receptors for partial structures of PGN; NOD1 and NOD2 recognize a diaminopimelic acid (DAP) containing peptide moiety [25,26] and a muramylpeptide moiety [27,28], respectively.

Previously, we demonstrated that PR3 activated human oral epithelial cells [29] and human gingival fibroblasts [30] via protease-activated receptor (PAR)-2. Furthermore, we demonstrated that proinflammatory cytokines induced production of PR3 in human oral epithelial cells, and the addition of murine anti-PR3 Abs to cytokine-primed oral epithelial cells induced the aggregation of PR3 followed by the activation of protease-activated receptor (PAR)-2, which resulted in remarkable secretion of IL-8 and MCP-1 [31]. Recently, we revealed that murine anti-human PR3 monoclonal Ab primed human monocytic THP-1 cells for enhanced activation upon stimulation by various TLR- and NOD-ligands [32]. In this study, we examined whether PR3-ANCA serum and purified IgG from WG patients were truly capable of activating human peripheral blood mononuclear cells (PBMCs) similary to the combination of murine anti-human PR3 Abs and THP-1 cells to release proinflammatory cytokines. If they are, the mechanism might be involved in the pathogenesis of ANCA-related chronic inflammatory diseases represented by WG.

## Methods

### Serum and immunoglobulin (Ig) samples

ANCA sera were obtained from 4 patients with WG at Tohoku University Hospital from Sep 1996 to Apr 2000. Sera from polyarteritis nodosa patients and aortitis syndrome patients were also obtained from totally 4 patients. The samples were immediately clarified by centrifugation, aliquoted, and frozen at -70°C until used. PR3-ANCA and MPO-ANCA titers were re-confirmed by EIA method by BML Co. (Sendai, Japan). All of the 4 ANCA sera used in this study were PR3-ANCA-positive and MPO-ANCA-negative specimens, and sera from polyarteritis nodosa and aortitis syndrome were PR3-ANCA-negative and MPO-ANCA-negative (Table 1). Normal serum from a healthy adult donor was used as a control. Purified Ig was prepared by absorption on protein G from the representative samples of PR3-ANCA sera and one sample of normal serum. The IgGs were negative for endotoxin in a Limulus amoebocyte lysate assay (sensitivity, 0.1 ng/ml). Prior to use, the IgGs were centrifuged to remove aggregates. For comparative purposes, a murine monoclonal anti-human PR3 Ab obtained from CLB (Amsterdam, The Netherlands) was used.

**Table 1:** Titers of PR3-ANCA and MPO-ANCA in serum from WG patients, polyarteritis nodosa patients, and aortitis syndrome patients used in this study.

No. (sample code) (bleeding date)	PR3-ANCA (U/ml)	MPO-ANCA (U/ml)
1. (Q9-40) (5/9/1996)	29.2	< 1.3
(R9-10) (9/27/1996)	19.1	< 1.3
(S9-31) (11/21/1996)	10.7	< 1.3
2. (R9-7) (9/27/1996)	3.8	< 1.3
(S9-29) (11/19/1996)	3.5	< 1.3
3. (S9-32) (11/21/1996)	18.8	< 1.3
4. (D10-41) (4/4/2000)	14.0	< 1.3
5. (M10-24) (2/3/2004)	< 1.3	< 1.3
6. (M10-28) (2/3/2004)	< 1.3	< 1.3
7. (G10-37) (5/15/2002)	< 1.3	< 1.3
8. (H10-51) (9/2/2002)	< 1.3	< 1.3

### Reagents

The synthetic MDP (MurNAc-L-Ala-D-isoGln) and synthetic *Escherichia coli*-type lipid A (LA-15-PP) were obtained from The Protein Research Foundation Peptide Institute (Osaka, Japan). Poly I:C was purchased from Sigma-Aldrich (St. Louis, MO, USA). Single-stranded (ss) Poly U was acquired from Invivogen (San Diego, CA, USA). A conventional CpG DNA, CpG DNA 1826 (TCCATGACGTTCCTGACGTT [CpG motif is underlined]), was purchased from SIGMA Genosys (Tokyo, Japan). The synthetic diacyl lipopeptide FSL-1 (*S*-[2,3-bis(palmitoyloxy)-(2*RS*)-propyl]-[*R*]-cysteinyl-GDPKHPKSF) was obtained from EMC microcollections (Tübingen, Germany). The synthetic desmuramylpeptides, a PGN fragment containing DAP, FK156 (D-lactoyl-L-Ala-γ-D-Glu-*meso*-DAP-Gly) [33] was supplied by Astellas Pharmaceutical Co. (Tokyo, Japan). Anti-TLR2 mAb (TL2.1) (mouse IgG), anti-TLR3 mAb (mouse IgG), anti-TLR4 mAb (HTA125) (mouse IgG), anti-TLR7 mAb (mouse IgG), and anti-TLR9 mAb (mouse IgG) were purchased from eBioscience (San Diego, CA, USA). Rabbit anti-NOD1 Ab (L-17) and rabbit anti-NOD2 Ab were obtained from Santa Cruz Biotechnology (Santa Cruz, CA, USA). Isotype control mouse IgG (MOPC-2) was purchased from Sigma-Aldrich. All other reagents were obtained from Sigma-Aldrich, unless otherwise indicated.

### Cells and cell culture

The human monocytic leukemia cell line THP-1, supplied by the Health Science Research Resources Bank (Osaka, Japan), was cultured in RPMI 1640 medium (Nissui Seiyaku, Osaka, Japan) with 10% heat-inactivated fetal calf serum (FCS) at 37°C in a humidified CO_2 _atmosphere. The THP-1 cells were maintained in a logarithmic phase of growth (2 × 10^5 ^to 2 × 10^6^) by passage every 3–4 days.

Human PBMCs were isolated from heparinized peripheral blood of healthy adult donors by Lympholyte-H (Cedarlane Laboratories, Hornby, Ontario, Canada) gradient centrifugation at 800 × *g *for 20 min at room temperature. The isolated PBMCs were washed three times with phosphate-buffered saline (PBS) and suspended in RPMI 1640 medium.

### Determination of cytokines in culture supernatants

The cells were collected and washed twice in PBS. They (2 × 10^5 ^cells per ml) were preincubated with anti-PR3 Ab, control Abs, PR-ANCA sera or control sera for 6 h. Subsequently, they were stimulated with or without a stimulant in RPMI 1640 medium with 10% FCS for 24 h in 96-well culture plates. The culture supernatants were collected and the levels of IL-6, IL-8, MCP-1 and TNF-α were determined with an ELISA kit (OptEIA ELISA Kits, BD Pharmingen). The concentrations of the cytokines in the supernatants were determined using the LS-PLATEmanager 2004 data analysis program (Wako Pure Chemical Industries, Osaka, Japan).

### Flow cytometry

Flow cytometric analyses were performed using a FACSCalibure cytometry (BD Biosciences, Mountain View, CA, USA). PBMCs were stained with anti-TLR2 Ab and anti-TLR4 Ab or control IgG at 4°C for 30 min, followed by fluorescein isothiocyanate (FITC)-conjugated secondary Ab (BioSource International, Camarillo, CA, USA) at 4°C for an additional 30 min. For TLR3, TLR7, TLR9, NOD1 and NOD2, intracellular staining was performed. Briefly, the cells were washed with staining buffer, fixed and permeabilized with BD Cytofix/Cytoperm solution (BD Biosciences) for 15 min at 4°C, and then incubated with anti-TLR3 Ab, anti-TLR7 Ab, anti-TLR9 Ab, anti-NOD1 Ab, anti-NOD2 Ab or control IgG for 30 min, followed by FITC-conjugated secondary Ab at 4°C for another 30 min. To calculate the percentage of positive cells, the baseline cursor was set at a channel that yielded < 2% of events positive with the isotype Ab control. Fluorescence to the right was counted as specific binding.

### Statistics

All experiments in this study were performed at least three times to confirm the reproducibility of the results. For most of the experiments, values are represented as the mean ± S.D. of triplicate assays. The significance of differences between two means was evaluated by one-way ANOVA using the Bonferroni or Dunnett method, and values of *P *< 0.05 were considered to be significant.

## Results

### Treatment with anti-PR3 Abs up-regulated the expression of TLR2, TLR3, TLR4, TLR7, TLR9, NOD1 and NOD2 in human PBMCs

**Figure 1:**
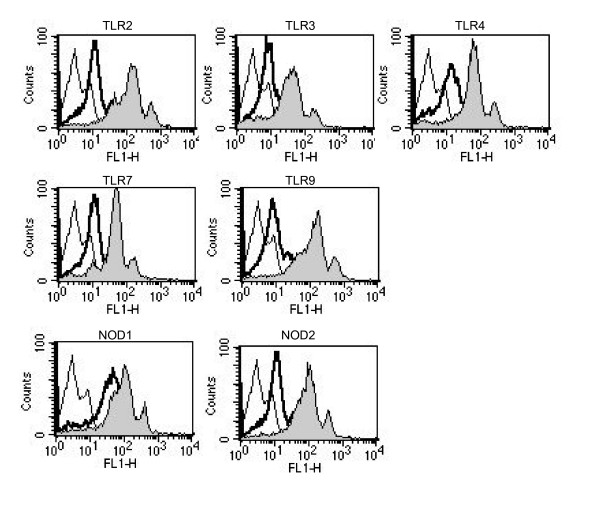
**Up-regulation of the expression of TLR2, TLR3, TLR4, TLR7, TLR9, NOD1, and NOD2 in human PBMCs in response to anti-PR3 Abs**. PBMCs were stimulated with murine anti-human PR3 Abs (1 μg/ml, filled graphs) or an isotype-matched control IgG (1 μg/ml, bold line). After 6 h of incubation, the expression of cell-surface TLR2 and TLR4 and intracellular TLR3, TLR7, TLR9, NOD1, and NOD2, was assessed by flow cytometry. The thin lined curve is the staining with a control Ab. The results presented are representative of four different experiments.

As mentioned above, we [32] recently reported that anti-PR3 Abs up-regulated the expression of various TLRs and NOD1/2 on human monocytic THP-1 cells, and endowed the cells with the ability to produce inflammatory cytokines at remarkable levels upon stimulation with various TLRs and NOD1/2 ligands. As it would be interesting to investigate whether similar effects induced by monoclonal anti-PR3 Abs are also observed with PBMCs, we examined the effects of anti-PR3 Abs on the expression of various TLRs and NOD1/2 in PBMCs. PBMCs constitutively expressed TLR2, 3, 4, 7, and 9, NOD1, and NOD2. The incubation of PBMCs with 1 μg/ml of the anti-PR3 Abs resulted in the up-regulated expression of these molecules (Fig. 1), consistent with the results obtained with the human monocytic cell line, THP-1. On the other hand, equal concentrations of an isotype-matched IgG were completely ineffective in this respect.

### ANCA serum amplified IL-8 production triggered by TLR and NOD ligands in human monocytic cells

**Figure 2:**
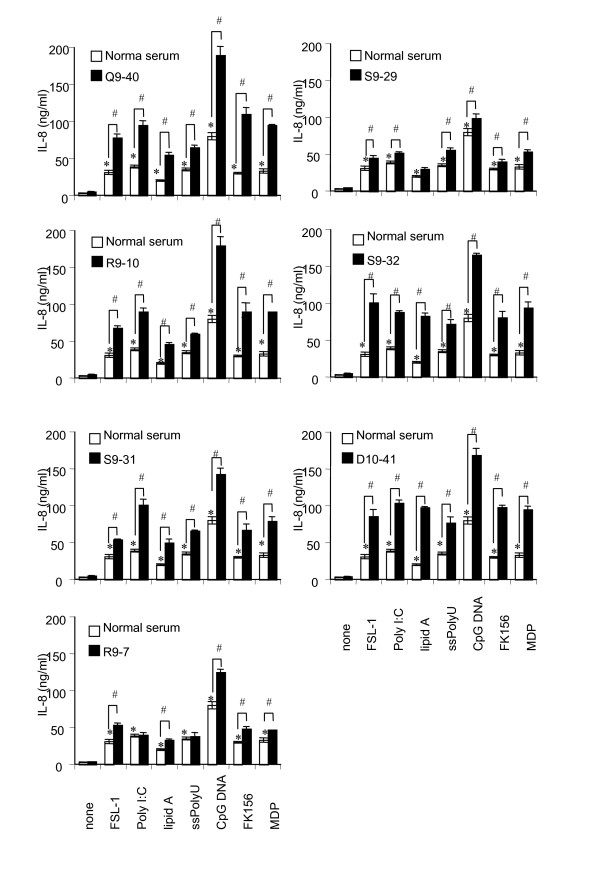
**Enhancement of TLR and NOD ligand-induced IL-8 production in human monocytic THP-1 cells preincubated with PR3-ANCA sera**. THP-1 cells (2 × 10^5 ^per ml) were preincubated for 6 h with 1:100 dilutions of PR3-ANCA sera from WG patients (Q9-40, R9-10, S9-31, R9-7, S9-29, S9-32, and D10-41) (closed bar) or with equal amounts of normal serum (sham incubation, open bar). Subsequently, THP-1 cells were challenged with FSL-1 (1 nM), poly I:C (1 μg/ml), lipid A (10 ng/ml), ssPoly U (10 μg/ml), CpG DNA (1 μM), FK156 (100 μg/ml), or MDP (100 μg/ml) for 18 h. IL-8 levels in the culture supernatants were determined by ELISA, and expressed as means ± S.D. *^,# ^Significantly different from sham-incubated THP-1 cells and from respective cultures stimulated with the respective ligands alone. The results are representative of three different experiments.

To further demonstrate the possible immunopathological properties of ANCA from WG patients, we examined whether the ANCA sera are capable of priming THP-1 cells similarly to murine anti-PR3 Ab. We obtained sera from patients with WG at Tohoku University Hospital, and carried out experiments using the sera and human Abs purified from the sera. The titers of PR3-ANCA and MPO-ANCA in serum were determined. All 7 sera (Q9-40, R9-7, R9-10, S9-29, S9-31, S9-32, and 10–41) from 4 patients were PR3-ANCA positive (> 3.0 titer) whereas none was MPO-ANCA positive (< 1.3 titer) (Table 1). Therefore, the seven PR3-ANCA-positive sera from WG patients were compared with normal serum. We examined the production of IL-8 upon stimulation with the respective ligands (TLR2-agonistic FSL-1, TLR3-agonistic poly I:C, TLR4-agonistic lipid A, TLR7-agonistic ssPoly U, NOD1-agonistic FK156, and NOD2-agonistic MDP) after priming with the sera. IL-8 production was significantly enhanced when THP-1 cells were incubated with WG patients sera (closed bar) as compared with normal serum (open bar) (Fig. 2). The priming effects of WG patients' sera correlated well with the PR3-ANCA titer; e.g. stronger priming effects were observed with Q9-40 (PR3-ANCA titer 29.2) and R9-10 (PR3-ANCA titer 19.1) and weaker priming effects were observed with R9-7 (PR3-ANCA titer 3.8) and S9-29 (PR3-ANCA titer 3.5). These results clearly indicated that PR3-ANCA in the sera from WG patients exerted a clear priming effect similar to murine monoclonal anti-human PR3 Abs in vitro.

### PR3-ANCA sera and purified IgG amplified the production of IL-6, IL-8, MCP-1, and TNF-α triggered by TLR and NOD ligands in PBMCs

**Figure 3:**
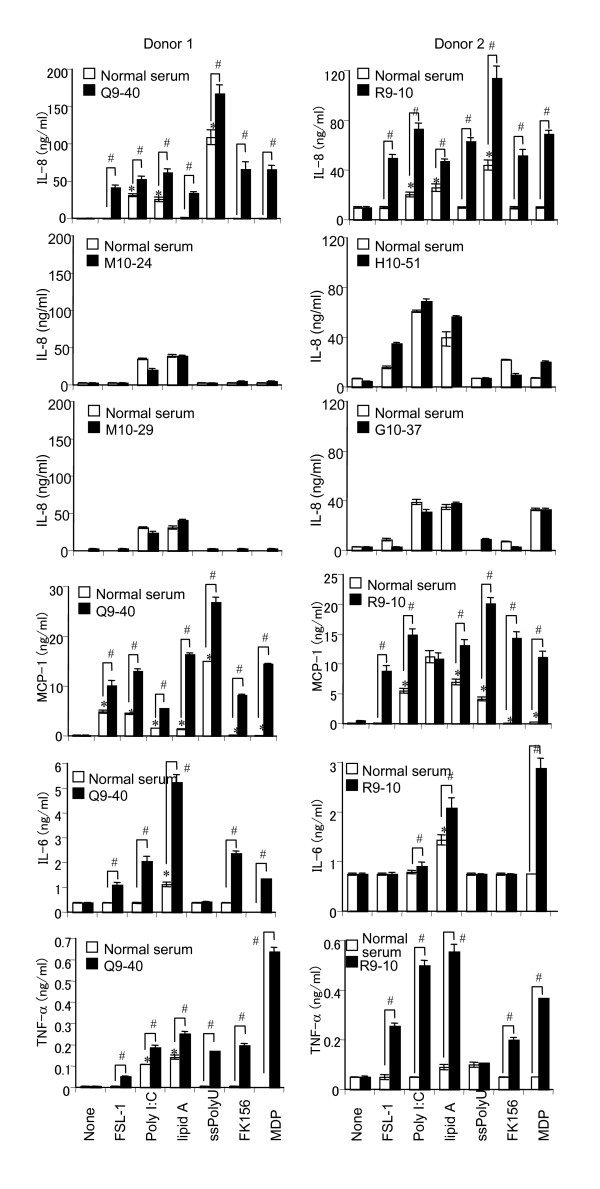
**Enhancement of TLR and NOD ligand-induced production of IL-8, MCP-1, IL-6, and TNF-α in human PBMCs preincubated with PR3-ANCA sera**. PBMCs (donor 1 and 2, 2 × 10^5 ^cells per ml) were preincubated for 6 h with a 1:20 dilution of PR3-ANCA sera (Q9-40 or R9-10 from WG patients or ANCA-negative vasculitis seum (M10-24, M10-29, H10-51, or G10-37) (closed bar), or with equal amounts of normal serum (sham incubation, open bar). Subsequently, PBMCs were challenged with FSL-1 (1 nM), poly I:C (1 μg/ml), lipid A (10 ng/ml), ssPoly U (10 μg/ml), FK156 (100 μg/ml), or MDP (100 μg/ml) for 18 h. The levels of IL-6, IL-8, MCP-1, and TNF-α in the culture supernatants were determined by ELISA, and expressed as means ± S.D. *^,# ^Significantly different from sham-incubated PBMCs and from respective cultures stimulated with the respective ligands alone. The results are representative of four different experiments.

**Figure 4:**
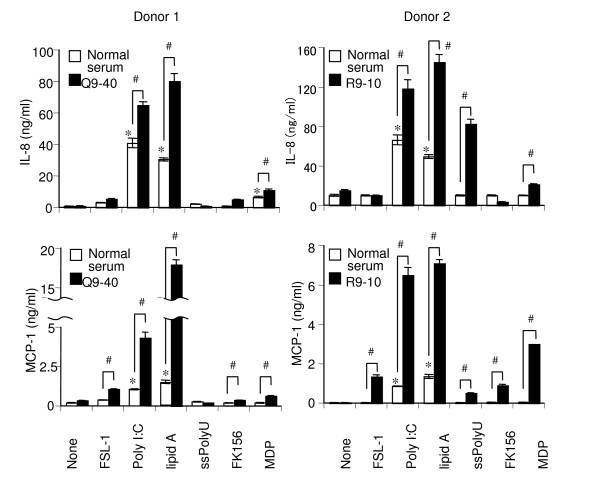
**Enhancement of TLR and NOD ligand-induced production of IL-8 and MCP-1 in human PBMCs preincubated with purified IgGs from PR3-ANCA sera**. PBMCs (donor 1 and 2, 2 × 10^5 ^cells per ml) were preincubated for 6 h with PR3-ANCA IgG (50 μg/ml, Q9-40 or R9-10) (closed bar) or with an equal amount of normal IgG (sham incubation, open bar). Subsequently, PBMCs were challenged with FSL-1 (1 nM), poly I:C (1 μg/ml), lipid A (10 ng/ml), ssPoly U (10 μg/ml), FK156 (100 μg/ml) or MDP (100 μg/ml) for 18 h. The levels of IL-8 and MCP-1 in the culture supernatants were determined by ELISA, and expressed as means ± S.D. *^,# ^Significantly different from sham-incubated PBMCs and from respective cultures stimulated with the respective ligands alone. The results are representative of four different experiments.

Next, we examined whether similar priming effects of PR3-ANCA sera are also observed with PBMCs. Consistent with the results in THP-1 cells (Fig. 2), PR3-ANCA sera (R9-40 and R9-10) promoted various TLRs and NOD1/2 ligand-induced secretion of IL-8. It must be noted here that the production of IL-6, MCP-1, and TNF-α in addition to IL-8 was also significantly up-regulated (Fig. 3). PBMCs were incubated with PR3-ANCA sera for various periods prior to being challenged with various TLRs and NOD1/2 ligands. Although the magnitude of the priming reaction differed among the respective ligands, similar kinetic patterns were observed and only a slight elevation was observed after a 2 h priming period and the ratio of the response increased until 6 h, when maximal enhancement was observed (data not shown). Thereafter, the priming effect decreased, although longer priming periods were still effective in this respect (data not shown). Although ANCA is autoantibodies characteristic of vasculitis diseases, PR3-ANCA and MPO-ANCA are known to be negative in some kinds of vasculitis such as polyarteritis nodosa and aortitis syndrome (Table 1). It must be noted here that four ANCA-negative vasculitis sera (M10-24, M10-29, H10-61, and G10-37) did not have any priming effects on PBMCs, contrary to two PR3-ANCA-positive sera (Q9-40 and R9-10) (Fig. 3). To further confirm the activity of PR3-ANCA other than the possible bioactive factor(s) in the sera, we purified IgG from PR3-ANCA sera by protein G. As shown in Fig. 4, purified IgG significantly up-regulated the production of IL-8 and MCP-1 in PBMCs.

## Discussion

Among ANCA, those targeting PR3 (PR3-ANCA) have a strong and specific association with WG [4]. Besides their significance as seromarkers, a pathogenic role has been proposed for these autoantibodies in relation to their capacity to activate leukocytes in vitro [9,10,12]. In the present study, an alternative approach was chosen to define the priming effect of ANCA against PR3 (PR3-ANCA) from WG patients on inflammatory leukocyte functions: human PBMCs were preincubated with substimulatory concentrations of PR3-ANCA and possible augmented cell activation by various microbial PAMPs was examined. As mentioned above, Hattar et al. [20] first reported that anti-PR3 Abs primed human leukocytes for the enhanced production of inflammatory cytokines upon stimulation with TLR4-agonistic LPS or TLR2-agonistic lipoteichoic acid in a CD14-dependent manner. In our previous study [32], we demonstrated that incubation with murine anti-PR3 Abs significantly up-regulated the expression of various TLRs (TLR2, 3, 4, 7, and 9, NOD1, and NOD2) in addition to CD14 in human monocytic THP-1 cells. In this context, however, Hattar et al. [20] found no increase in the expression of TLR2 and TLR4 by FACS analysis after treatment with anti-PR3 Abs, and their priming effects were CD14-dependent, but TLR2- and TLR4-independent. It is unclear why they did not find an increase in the expression of TLR. The difference in staining methods may be one reason; in their study, the cells were directly stained with PE-labeled Abs targeting TLR2 or TLR4, whereas in our study the cells were stained with various TLR or NOD Abs, followed by a FITC-conjugated secondary Ab.

In our previous study [32], we examined the priming effects of murine monoclonal anti-human PR3 Abs on human monocytic THP-1 cells. We were interested in whether similar effects would be observed with human PBMCs and with serum and purified Ig isolated from WG patients and control sera. In this study, we demonstrated that PR3-ANCA serum from WG patients up-regulated the expression of various TLRs and NODs in human PBMCs (Fig. 1) and primed PBMCs to produce proinflammatory cytokines such as IL-6, IL-8, MCP-1, and TNF-α via TLRs and NODs (Figs. 2 and 3). We solely used chemically synthesized PAMPs, because natural microbial cell surface preparations are inevitably contaminated with minor bioactive components that might have confused the results. Therefore, these results clearly indicated that PR3-ANCA primed TLR2-, 3-, 4-, 7-, and 9-, NOD1-, and 2-dependent cell activation in PBMCs. Although ANCA is autoantibodies characteristic of vasculitis diseases, PR3-ANCA and MPO-ANCA are known to be negative in some kinds of vasculitis such as polyarteritis nodosa and aortitis syndrome (Table 1). It must be noted here that four ANCA-negative vasculitis sera did not have any priming effects on PBMCs in PBMCs, contrary to two ANCA-positive sera (Fig. 3). Purified IgG from WG serum also significantly primed PBMCs to produce proinflammatory cytokines (Fig. 4). These findings certainly strengthen our conclusion and the relevance of our work to the human situation.

It is conceivable that microbial components exhibit powerful immuno-adjuvant activities through the activation of antigen-presenting cells via TLR and NOD pathways against various antigens, including autoantigens, which in turn might induce severe autoimmune diseases. In fact, in animal models, NOD2-agonistic MDP induces autoimmune diseases such as experimental encephalomyelitis, orchitis, uveoretinitis, thyroiditis, and polyarthritis [34]. In contrast, our study showed a completely different model for the possible induction of autoimmune diseases by microbial components. Namely, microbial TLR- and NOD-ligands possibly induced tissue destruction through excessive inflammatory responses by triggering the activation of PR3-ANCA-primed cells. The background for the present experimental rationale is based on the fact that the disease activity in WG, which is paralleled by a rising PR3-ANCA titer [4,8], appears to be triggered by microbial infections [9,35-37]. As suggested by Condliffe et al. [38] and Hallett and Lloyds [39], in vivo, the response of leukocytes to microbial depends on the state of cellular activation, varying from "dormant" via "primed" to "fully activated". Priming is a key mechanism involved in the regulation of the leukocyte-dependent host defense. Although they do not directly activate neutrophil and monocyte functions, priming agents induce a "sensitization" of the leukocytes for subsequent stimulation with naturally occurring agonists, such as microbe-derived products. In addition to the important roles of ANCA in the regulation of inflammatory leukocyte functions, the phenomenon of leukocyte priming by PR3-ANCA may also be relevant to the pathogenesis of inflammation. Indeed, in active WG, neutrophils and monocytes display a phenotype attributable to a state of cellular preactivation with the enhanced surface expression of activation markers such as CD11b and CD64 [11,40].

PR3-ANCA, being only weak direct activators of monocytes and neutrophils to release cytokines per se, exert a definite priming effect on these leukocytes, enhancing their responsiveness to secondary stimulation with microbial PAMPs. The up-regulation of various pattern recognition receptors, including TLRs and NODs, acting as respective PAMPs, was characterized as one mechanism underlying the ANCA-elicited priming response. Such cooperation between PR3-ANCA and microbial PAMPs may well trigger exacerbations of disease activity during infections and contribute to the persistence of inflammatory lesions, which might be a novel model for the pathogenesis of autoimmune diseases.

## Conclusion

In conclusion PR3-ANCA prime human mononuclear cells to produce cytokines upon stimulation with various microbial components by up-regulating the TLR and NOD signaling pathway, and these mechanisms *may partially *participate in inflammatory process in WG.

## Abbreviations

ANCA: anti-neutrophil cytoplasmic antibodies; Abs: antibodies; WG: Wegener's granulomatosis; PR3: proteinase 3; MPO: myeloperoxidase; TNF: tumor necrosis factor; IL: interleukine; MCP: monocyte chemoattractant protein; LPS: lipopolysaccharide; Ig: immunoglobulin; PAMPs: pathogen-associated molecular patterns; PGNs: peptidoglycans; TLR: Toll-like receptor; MDP: muramyldipeptide; DAP: diaminopimelic acid; PBMCs: peripheral blood mononuclear cells; FCS: fetal calf serum; PBS: phosphate-buffered saline; FITC: fluorescein isothiocyanate.

## Competing interests

The authors declare that they have no competing interests.

## Authors' contributions

AU carried out the flow cytometry and was involved in the design of the study and drafting the manuscript. TS, AI and SY carried out cell culture experiments and was involved in drafting the manuscript. All authors read and approved the final manuscript.
